# Brachial artery reactivity and vascular reactive hyperemia for preoperative anaesthesia risk assessment – an observational study

**DOI:** 10.1186/1471-2253-14-47

**Published:** 2014-06-21

**Authors:** Robert Schier, Volker Schick, Ashley Amsbaugh, Jorge Aguilar, Mike Hernandez, Reza J Mehran, Bernhard Riedel, Jochen Hinkelbein

**Affiliations:** 1 Department of Anaesthesiology and Intensive Care Medicine, University Hospital of Cologne, Cologne, Germany; 2 Department of Anesthesiology & Pain Medicine, The University of Texas M. D. Anderson Cancer Center, Houston, TX, USA; 3 Department of Biostatistics, The University of Texas M. D. Anderson Cancer Center, Houston, TX, USA; 4 Department of Thoracic and Cardiovascular Surgery, The University of Texas M. D. Anderson Cancer Center, Houston, TX, USA; 5 Department of Anaesthesia and Pain Medicine, Peter MacCallum Cancer Centre and The University of Melbourne, Melbourne, Australia

**Keywords:** Vascular function, Non-invasive diagnostic tool, Preoperative anesthesia assessment

## Abstract

**Background:**

Non-invasive measures of vascular reactivity have emerged to refine cardiovascular risk. However, limited data exists investigating vascular reactivity as a preoperative diagnostic tool for anesthesiologists. In this study, we compare the utility of two non-invasive techniques, Brachial Artery Reactivity Testing (BART) and Digital Thermal Monitoring (DTM), as surrogates for measuring vascular reactivity.

**Methods:**

Following IRB approval, 26 patients scheduled for major thoracic surgery (e.g. esophagectomy and pneumonectomy) were studied prospectively. BART [Flow mediated dilation (FMD) and Peak flow velocity (PFV)] and DTM [Temperature rebound (TR%)] were performed preoperatively at baseline using 5 minute blood pressure cuff occlusion of the upper arm. Statistical summaries were provided for the comparison of BART and DTM with select patient characteristics, and correlations were used to investigate the strength of the relationship between BART and DTM measurements.

**Results:**

Patients preoperatively diagnosed with hyperlipidemia were associated with lower FMD% values {Median (Range): 14.8 (2.3, 38.1) vs. 6.2 (0.0, 14.3); p = 0.006}. There were no significant associations between BART and DTM techniques in relation to cardiovascular risk factors or postoperative complications.

**Conclusion:**

Our study suggests that impaired vascular reactivity as measured by BART is associated with the incidence of hyperlipidemia. Also, using a novel technique such as DTM may provide a simpler and more accessible point of care testing for vascular reactivity in a perioperative setting. Both non-invasive techniques assessing vascular function warrant further refinement to better assist preoperative optimization strategies aimed at improving perioperative vascular function.

## Background

Postoperative cardiovascular events are responsible for a substantial proportion of the morbidity and mortality of patients undergoing non-cardiac surgery [1]. With the availability of many practical lifestyle and pharmacological interventions that can be directed at patients with an increased risk of vascular complications (i.e. smoking cessation, exercise or statin therapy [2]), early identification of these patients has long been of interest to clinicians in order to prevent postoperative complications [3,4]. Recently, non-invasive measures of vascular function have gained increasing importance, as they are used in addition to standard cardiovascular disease (CVD) risk factors in an attempt to refine risk stratification in patients undergoing non-cardiac surgery [5]. However, these measures are technically challenging and not ideal for clinical purposes, especially if serial measurements are required [6].

Brachial Artery Reactivity Testing (BART) is a non-invasive technique that has been established over the past few years for the evaluation of preclinical disease states geared at improving vascular function with targeted specific interventions and risk factor modifications [7]. Unfortunately, assessing flow-mediated dilation (FMD) and peak flow velocity (PFV) requires technical expertise and ultrasound equipment. Hence, it is restricted to the setting of a vascular laboratory. A novel technique that is currently under investigation in clinical trials is Digital Thermal Monitoring (DTM). This non-invasive method is currently under evaluation for the assessment of peripheral vascular function and improvement of cardiovascular risk assessment [8,9]. Similar to FMD measured by ultrasound, this method measures changes in skin blood flow induced by reactive hyperemia by utilizing a temperature rebound (TR) [10]. Controversy remains whether there is a correlation present between macrovascular- measured via ultrasound in the brachial artery (FMD), and predominantly microvascular hyperemic responses- measured at the fingertip via (DTM) or pulse waveform analysis.

A recent study investigating pulse waveform analysis refuted the claims that large (macrovascular) and small (microvascular) arterial stiffness are surrogate measures for sonographic assessments of brachial FMD [11].

The aim of this study was to evaluate two non-invasive techniques (BART and DTM) for the preoperative assessment of vascular function. Outcome parameters consisted of the two tested techniques with cardiovascular risk factors such as: hypertension, diabetes, hyperlipidemia, obesity, smoking and the incidence of postoperative complications.

## Methods

The study was approved by the local Ethics Committee (The University of Texas M.D. Anderson Cancer Center, approval number 2003–0434) and all patients signed an informed consent. Thirty patients undergoing major thoracic surgery (lobectomy, pneumonectomy, esophagectomy) were eligible consented for this prospective observational study. We chose major thoracic surgery patients due to their high incidence of cardiovascular risk factors and postoperative cardiovascular complications (e.g. postoperative morbidity of 15-36% and mortality of 4.8-10.9% following pneumonectomy) [4,12].

Four patients had to be excluded due to low baseline fingertip temperature at the start of the DTM assessment. Therefore, a total of twenty-six consecutive patients with thoracic cancer (i.e. pulmonary, esophageal cancer) participated in this study. Other exclusion criteria included: being under 18 years of age, pregnant, the presence of a recent or unstable myocardial infarction, cerebrovascular accident, pulmonary embolus, deep vein thrombosis, or any condition that deemed the patient unsatisfactory for surgery.

### Brachial Artery Reactivity Testing (BART)

Patients were tested preoperatively within one week of undergoing surgery. With patients in a fasting state and in a supine position, ultrasound measurements of the brachial artery were performed in a quiet, dark room under stable temperature conditions (25°C). Resting blood pressure was measured by placing a blood pressure cuff on the right forearm. One operator blinded to the study patients’ medical history obtained right arm brachial artery ultrasound images (Philips Excelera, Andover, MA 01810, USA) at baseline and at 30, 60, 90 and 120 seconds after a 5 minute occlusion with 50 mmHg above systolic blood pressure. Post-occlusion FMD (in mm) and PFV (in cm/sec) were expressed in percent diameter and flow increase- absolute values were also recorded. Patients were grouped into low, medium, and high tertiles according to the increase of FMD and PFV values following arm occlusion.

### Digital Thermal Monitoring (DTM)

**Figure 1:**
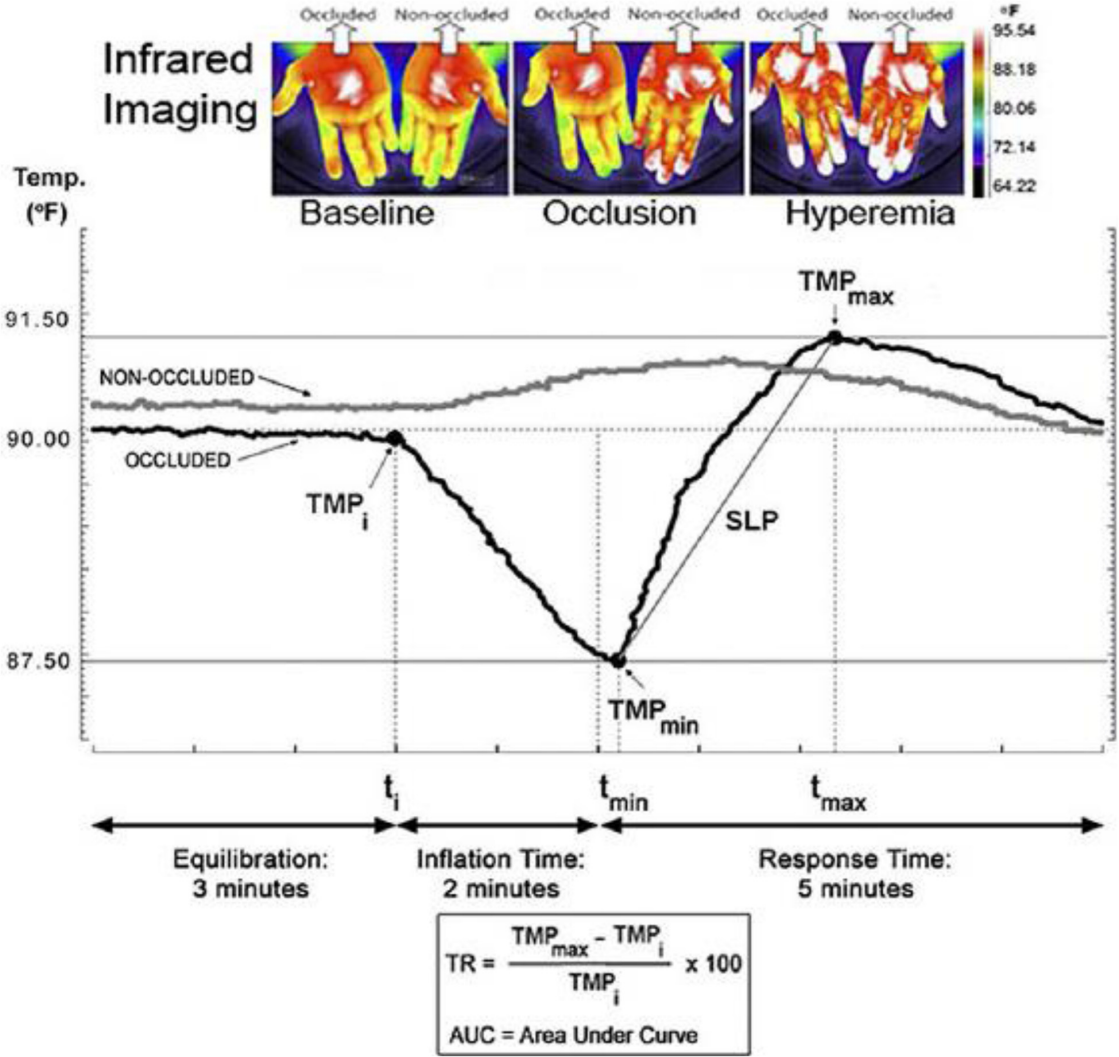
**Representative example of a temperature – time trace within the DTM assessment.**

DTM measurements were performed subsequent to FMD measurements, with at least 10 minutes between each procedure. After an overnight fast and abstinence from tobacco, alcohol, and caffeine, patients, in a supine position, were tested in a quiet, dark room under stable temperature conditions (25°C). DTM was measured using the VENDYS® 5000BC device (Endothelix Inc., Houston, TX, USA), which utilizes a computer-based thermometry system (0.01°C thermal resolution) and two fingertip thermocouple probes attached to the index finger of each hand (left: occlusion; right: control). Standard sphygmomanometer cuffs were placed on each upper arm (left: occlusion; right: control). After a period of stabilization of basal skin temperature, the right cuff was rapidly inflated to ≥ 50 mmHg above systolic blood pressure (measured on control arm). After a 5 minute arm occlusion the cuff was then rapidly deflated and a temperature rebound (TR%) was measured in the right index finger in response to the invoked reactive hyperemia for 5 minutes. A representative example of a temperature/time trace and a list of the measured DTM parameters are shown in the Figure 1 and Table 1.

**Table 1:** List of DTM parameters

**Temperature (T) in Celsius (°C)**	
TMPi	Initial fingertip temperature at cuff inflation
TMPmin	Lowest temperature (nadir) observed after cuff inflation
TMPmax	Highest temperature observed after cuff deflation
**Time (t) in seconds (s)**	
Ti	Time to cuff inflation
Tmin	Time of TMPmin
Tmax	Time of TMPmax
**Derived parameters**	
TF	Temperature Fall (°C; TMPmax – TMPi)
TR°C	Temperature Rebound (°C; TMPmax - TMPi)
TR%	Temperature Rebound (%; (TR°C/TMPi)*100)
NP	Nadir to Peak (°C; TMPmax – TMPmin)
SLP	Slope (°C/s; NP/Time to Reach TR)

### Perioperative risk assessment

Patient data was obtained using the clinical database (ClinicStation™). In addition, patients’ perioperative risk profile was assessed using established pre-operative risk scores from the American Society of Anesthesiologists (ASA) Physical Status Classification System, the Modified Lee Cardiac Risk Index, and the American Heart Association/American College of Cardiology (ACC/AHA) Risk Score). Two blinded investigators collected data about perioperative events according to standardized criteria. Patients were monitored during their hospital stay, approximately 30 days, and 6 months after surgery. Postoperative events were evaluated according to predefined adverse event categories and definitions (Additional file 1).

### Statistical analysis

Descriptive statistics consisting of means and standard deviations, medians and ranges, or frequencies and percents were used to summarize our patients’ data. Based on a previous study [13], we assumed a change in brachial artery diameter before and after forearm ischemia of 17.8 ± 10.9% (4.0 ± 0.6 mm at rest vs. 4.7 ± 0.6 mm at 60 sec.) after cuff deflation would be a detectable difference observable with 26 patients (α = 0.05 and power >0.8, two-sided paired *t*-test). For purposes of conducting exploratory analyses, select patient variables were converted to categorical variables by splitting the data at the median value thus creating a variable with two levels. Continuous measures of FMD%, PFV%, TR%, and TR(°C) were then compared between the levels of these patient characteristics using *t*-test or the Wilcoxon rank-sum test, if more appropriate. A correlation analysis was used to evaluate the bivariate relationship between BART and DTM parameters. For the DTM measurements, we excluded tests with a baseline fingertip temperature below 27°C in reference to a previous study indicating that it would provide an unreliable temperature rebound (TR) value due to predisposed vasoconstriction in a relatively cold finger [10].

Multivariate analyses were not undertaken due to the study’s small sample size. A p-value less than 0.05 was considered statistically significant.

## Results

Clinical characteristics of the subjects (n = 26; age, 59 ± 10 years; range, 28–78) are shown in Table 2. Patients with hyperlipidemia were associated with lower FMD% values {Median (Range): 14.8 (2.3, 38.1) vs. 6.2 (0.0, 14.3); p = 0.006, Table 3). Patients with other preoperative cardiac risk factors (smoking, obesity, hypertension, diabetes, preoperative chemo-/radiation therapy) or classified with an ASA, Lee Cardiac Risk Index or ACC/AHA score greater than 2 did not differ in FMD%, PFV% and TR%-values when compared to the rest of the study group (Tables 3 and 4). Preoperative cardiovascular medication (statin, ACE-inhibitor, AT2-inhibitor or beta-blocker therapy) did not have an impact on BART or DTM values (Tables 3 and 4). Neither BART nor DTM were significantly related to the incidence of postoperative complication (Table 5). Neither FMD% nor PFV% were significantly correlated with TR% (Table 6). However, postoperative complications were associated with a longer length of hospital stay (29.7 ± 31.7 vs. 6.0 ± 4.3 days; p = 0.002).

**Table 2:** Summary of patient characteristics

**Characteristics**	**All patients N = 26**
Age in years	
Mean ± SD, (Range)	59.3 ± 10.4, (28, 78)
Female sex	9 (35%)
Caucasian	20 (77%)
Length of Hospital Stay in days	
Mean ± SD, (Range)	11.5 ± 17.8, (2, 77)
Length of ICU Stay in days	
Mean ± SD, (Range)	4.0 ± 14.4, (0, 63)
ASA	
2	2 (8%)
3	24 (92%)
Lee Cardiac Risk Index	
2	25 (96%)
3	1 (4%)
ACC/AHA Risk Score	
≤ 1	23 (89%)
> 1	3 (11%)
Chemotherapy	
No	13 (50%)
Yes	13 (50%)
Radiation	
No	15 (58%)
Yes	11 (42%)
Obesity^*^	
No	16 (61%)
Yes	10 (39%)
Current/Former Smoker	
No	9 (35%)
Yes	17 (65%)
Coronary Artery Disease	
No	26 (100%)
Yes	0 (0%)
Hypertension	
No	12 (46%)
Yes	14 (54%)
Diabetes mellitus	
No	23 (88%)
Yes	3 (12%)
Dyslipidemia	
No	17 (65%)
Yes	9 (35%)
Statin Therapy	
No	20 (77%)
Yes	6 (23%)
Beta-blocker Therapy	
No	20 (77%)
Yes	6 (23%)
ACE-Inhibitor Therapy	
No	23 (88%)
Yes	3 (12%)
AT2-Inhibitor Therapy	
No	25 (96%)
Yes	1 (4%)

ASA = American Society of Anesthesiologists physical status classification system; AT2 = Antagonist.

*BMI >30 kg/m^2^.

**Table 3:** Summary of patient characteristics by BART parameters

	**FMD%**				**PFV%**			
**Characteristics**	**N**	**Mean ± SD**	**Median (Range)**	p-value*	**N**	**Mean ± SD**	**Median (Range)**	p-value*
Age								
<60	15	12.8 ± 9.0	12.5 (0, 38.1)	0.795	15	103.1 ± 64.2	90 (10, 291)	0.243
>60	11	11.9 ± 9.5	11.1 (0, 29.7)		11	78.0 ± 50.6	81 (21, 175)	
Sex								
Male	17	11.3 ± 7.4	11.1 (0, 29.7)	0.418	17	102.0 ± 65.3	90 (10, 291)	0.346
Female	9	14.6 ± 11.6	14.8 (0, 38.1)		9	74.4 ± 42.6	60 (21, 139)	
Smoker								
No	9	11.0 ± 11.9	10.3 (0, 38.1)	0.293	9	82.9 ± 46.9	96 (10, 139)	0.979
Yes	17	13.2 ± 7.4	14.3 (0, 29.7)		17	97.5 ± 65.4	85 (21, 291)	
Length of stay								
<5	12	12.9 ± 8.2	12.2 (0, 29.7)	0.554	12	76.9 ± 40.1	83.5 (21, 139)	0.304
>5	14	12.0 ± 9.9	10.45 (0, 38.1)		14	105.8 ± 70.2	93.5 (10, 291)	
Obesity								
No	16	12.9 ± 10.2	11.5 (0, 38.1)	0.958	16	87.6 ± 38.1	88.5 (10, 145)	1.00
Yes	10	11.7 ± 7.3	12.3 (0, 22.2)		10	100.3 ± 84.6	76 (21, 291)	
Chemotherapy								
No	13	15.8 ± 9.3	11.9 (6.2, 38.1)	0.117	13	75.0 ± 40.2	85 (10, 139)	0.191
Yes	13	9.1 ± 7.7	8.9 (0, 20.5)		13	109.9 ± 70.6	98 (30, 291)	
Radiotherapy								
No	15	15.4 ± 9.5	12.5 (0, 38.1)	0.058	15	78.4 ± 46.4	85 (10, 175)	0.243
Yes	11	8.4 ± 6.8	8.9 (0, 18.9)		11	111.6 ± 70.7	98 (30, 291)	
ASA Score								
2	2	11.4 ± 7.4	11.45 (6.2, 16.7)	**	2	29.5 ± 27.6	29.5 (10, 49)	**
3	24	12.5 ± 9.3	11.5 (0, 38.1)		24	97.7 ± 58.2	89.5 (21, 291)	
Lee Cardiac Risk Index								
2	25	12.7 ± 9.1	11.9 (0, 38.1)	**	25	95.8 ± 57.8	89 (21, 291)	**
3	1	6.2 ± NA	6.2 (6.2, 6.2)		1	10.0 ± NA	10 (10, 10)	
ACC/AHA Risk Score								
≤2	23	12.6 ± 9.3	11.9 (0, 38.1)	**	23	94.1 ± 57.2	89 (21, 291)	**
>2	2	6.8 ± 0.9	6.85 (6.2, 7.5)		2	32.0 ± 31.1	32 (10, 54)	
Diabetes Mellitus								
No	23	12.4 ± 9.4	11.9 (0, 38.1)	**	23	91.4 ± 57.6	88 (21, 291)	**
Yes	3	12.3 ± 7.4	10.3 (6.2, 20.5)		3	100.3 ± 83.6	116 (10, 175)	
Hypertension								
No	12	16.1 ± 9.1	13.4 (7.5, 38.1)	0.089	12	90.0 ± 33.9	89 (26, 145)	0.719
Yes	14	9.3 ± 8.0	8.25 (0, 22.2)		14	94.6 ± 75.6	85.5 (10, 291)	
Hyperlipidemia								
No	17	15.6 ± 9.0	14.8 (2.3, 38.1)	0.006	17	90.4 ± 45.8	89 (21, 175)	0.571
Yes	9	6.4 ± 5.5	6.2 (0, 14.3)		9	96.4 ± 81.7	82 (10, 291)	
Statin Therapy								
No	20	13.8 ± 9.6	13.4 (0, 38.1)	0.120	20	90.7 ± 44.0	89.5 (21, 175)	0.429
Yes	6	7.9 ± 5.2	8 (0, 14.3)		6	98.3 ± 100.2	81.5 (10, 291)	
ACE-Inhibitor Therapy								
No	23	13.2 ± 9.3	12.5 (0, 38.1)	**	23	93.4 ± 59.7	88 (21, 291)	**
Yes	3	6.5 ± 3.7	6.2 (3, 10.3)		3	85.0 ± 65.3	116 (10, 129)	
AT2-Inhibitor Therapy								
No	25	12.2 ± 9.2	11.1 (0, 38.1)	**	25	94.2 ± 59.6	89 (10, 291)	**
Yes	1	16.7 ± NA	16.7 (16.7, 16.7)		1	49.0 ± NA	49 (49, 49)	
Beta-Blocker Therapy								
No	20	12.9 ± 9.8	12.2 (0, 38.1)	0.670	20	94.0 ± 61.3	89.5 (21, 291)	0.808
Yes	6	10.9 ± 6.1	8.9 (5.1, 20.5)		6	87.5 ± 55.9	85 (10, 175)	

*p-value from Wilcoxon rank-sum test.

**p-value not provided if ≤ 3 observations in a group.

**Table 4:** Summary of Patient Characteristics by DMT Parameters

	**TR%**				**TR (°C)**			
**Characteristics**	**N**	**Mean ± SD**	**Median (Range)**	**p-value**^**002A**^	**N**	**Mean ± SD**	**Median (Range)**	p-value^*^
Age								
<60	15	-0.17 ± 0.95	-0.24 (-1.95, 2.01)	0.392	15	-0.05 ± 0.31	-0.08 (-0.63, 0.66)	0.421
>60	11	-0.01 ± 1.44	0.38 (-2.69, 2.02)		11	-0.02 ± 0.47	0.13 (-0.91, 0.63)	
Sex								
Male	17	0.19 ± 1.02	0.04 (-1.7, 2.02)	0.125	17	0.06 ± 0.33	0.01 (-0.54, 0.66)	0.112
Female	9	-0.66 ± 1.26	-0.51 (-2.69, 1.09)		9	-0.22 ± 0.42	-0.18 (-0.91, 0.37)	
Smoker								
No	9	0.42 ± 1.09	0.51 (-1.6, 2.02)	0.090	9	0.13 ± 0.35	0.17 (-0.53, 0.63)	0.100
Yes	17	-0.38 ± 1.13	-0.19 (-2.69, 2.01)		17	-0.12 ± 0.37	-0.06 (-0.91, 0.66)	
Length of Stay								
<5	12	-0.18 ± 1.39	0.19 (-2.69, 2.01)	0.797	12	-0.06 ± 0.46	0.07 (-0.91, 0.66)	0.758
>5	14	-0.04 ± 0.98	-0.16 (-1.95, 2.02)		14	-0.02 ± 0.31	-0.05 (-0.63, 0.63)	
Obesity								
No	16	-0.27 ± 1.28	-0.14(-2.69, 2.01)	0.429	16	-0.09 ± 0.42	-0.05 (-0.91, 0.66)	0.493
Yes	10	0.17 ± 0.93	-0.08 (-1.43, 2.02)		10	0.05 ± 0.30	-0.03 (-0.47, 0.63)	
Chemotherapy								
No	13	-0.27 ± 0.93	-0.04 (-1.7, .79)	0.663	13	-0.09 ± 0.30	-0.01 (-0.54, 0.26)	0.663
Yes	13	0.06 ± 1.37	-0.12 (-2.69, 2.02)		13	0.01 ± 0.45	-0.04 (-0.91, 0.66)	
Radiotherapy								
No	15	-0.57 ± 1.11	-0.24 (-2.69, .79)	0.052	15	-0.19 ± 0.36	-0.08 (-0.91, 0.26)	0.052
Yes	11	0.54 ± 0.93	0.26 (-.41, 2.02)		11	0.17 ± 0.29	0.09 (-0.14, 0.66)	
ASA Score								
2	2	-0.46 ± 1.37	-0.46 (-1.43, .51)	**	2	-0.15 ± 0.45	-0.15 (-0.47, 0.17)	**
3	24	-0.07 ± 1.17	-0.08 (-2.69, 2.02)		24	-0.03 ± 0.38	-0.03 (-0.91, 0.66)	
Lee Cardiac Risk Index								
2	25	-0.13 ± 1.18	-0.12 (-2.69, 2.02)	**	25	-0.05 ± 0.38	-0.04 (-0.91, 0.66)	**
3	1	0.51 ± NA	0.51(.51, .51)		1	0.17 ± NA	0.17 (0.17, 0.17)	
ACC/AHA Risk Score								
≤2	23	-0.13 ± 1.23	-0.19 (-2.69, 2.02)	**	23	-0.05 ± .4	-0.06 (-0.91, 0.66)	**
>2	2	0.23 ± 0.39	0.24 (-.04, .51)		3	0.04 ± .11	-0.01 (-0.04, 0.17)	
Diabetes Mellitus								
No	23	-0.12 ± 1.23	-0.04 (-2.69, 2.02)	**	23	-0.04 ± 0.40	-0.01 (-0.91, 0.66)	**
Yes	3	0.05 ± 0.40	-0.12 (-.24, .51)		3	0.02 ± 0.13	-0.04 (-0.08, 0.17)	
Hypertension								
No	12	-0.45 ± 1.04	-0.25 (-2.69, .79)	0.165	12	-0.15 ± 0.35	-0.08 (-0.91, 0.26)	0.181
Yes	14	0.20 ± 1.21	0.19 (-1.95, 2.02)		14	0.06 ± 0.39	0.07 (-0.63, 0.66)	
Hyperlipidemia								
No	17	-0.23 ± 1.09	-0.12 (-2.69, 2.02)	0.467	17	-0.08 ± 0.36	-0.04 (-0.91, 0.63)	0.435
Yes	9	0.14 ± 1.32	0.38 (-1.95, 2.01)		9	0.04 ± 0.42	0.13 (-0.63, 0.66)	
Statin Therapy								
No	20	-0.24 ± 1.14	-0.16 (-2.69, 2.02)	0.301	20	-0.08 ± 0.36	-0.05 (-0.91, 0.63)	0.273
Yes	6	0.34 ± 1.23	0.45 (-1.6, 2.01)		6	0.12 ± 0.41	0.15 (-0.53, 0.66)	
ACE-Inhibitor Therapy								
No	23	-0.13 ± 1.23	-0.12 (-2.69, 2.02)	**	23	-0.05 ± 0.40	-0.04 (-0.91, 0.66)	**
Yes	3	0.10 ± 0.38	0.04 (-0.24, 0.51)		3	0.03 ± 0.13	0.01 (-0.08, 0.17)	
AT2-Inhibitor Therapy								
No	25	-0.05 ± 1.15	-0.04 (-2.69, 2.02)	**	25	-0.02 ± 0.37	-0.01 (-0.91, 0.66)	**
Yes	1	-1.43 ± NA	-1.43 (-1.43, -1.43)		1	-0.47 ± NA	-0.47 (-0.47, -0.47)	
Beta-Blocker Therapy								
No	20	-0.22 ± 1.23	-0.07 (-2.69, 2.02)	0.503	20	-0.08 ± 0.40	-0.03 (-0.91,0 .63)	0.465
Yes	6	0.30 ± 0.89	-0.08 (-0.31, 2.01)		6	0.10 ± 0.29	-0.03 (-0.10, 0.66)	

*p-value from Wilcoxon rank-sum test.

**p-value not provided if ≤ 3 observations in a group.

**Table 5:** BART and DTM measures by complication

	Complications*		
	**Yes (N = 6)**	**No (N = 20)**	**p-value**
**BART Parameters**			
FMD			
Mean ± SD	6.6 ± 4.4	14.2 ± 9.4	0.07
Median (Range)	8.2 (0, 11.1)	14.3 (0, 38.1)	
PFV			
Mean ± SD	84.7 ± 39.0	94.8 ± 64.5	0.72
Median (Range)	85.0 (31, 138)	89.0 (10, 291)	
**DTM Parameters**			
TR%			
Mean ± SD	0.38 ± 1.20	-0.25 ± 1.20	0.25
Median (Range)	0.17 (-1.24, 2.02)	-0.20 (-2.70, 2.00)	
TR (°C)			
Mean ± SD	0.12 ± 0.35	-0.08 ± 0.38	0.27
Median (Range)	0.06 (-0.38, 0.63)	-0.06 (-0.91, 0.66)	

*Postoperative Cardiac, Pulmonary, Wound Healing and Surgical Events.

**Table 6:** Correlation of BART vs. DTM Parameters

**BART (N = 26)**	**DTM (N = 26)**	**Pearson Correlation**	
**Parameters**	**Parameters**	**r**	**p-value**
FMD%	TR%	-0.36	0.07
	TR (°C)	-0.36	0.07
PFV%	TR%	-0.10	0.63
	TR (°C)	-0.10	0.63

## Discussion

The aim of our study is to evaluate the utility of BART and DTM, two different techniques with similar endpoints, for the preoperative assessment of vascular function in patients presenting at the anesthesia clinic. Both techniques utilize the principle of indirectly measuring reactive hyperemia in response to arm occlusion. Our study indicated that both techniques were comparable with respect to preoperative cardiovascular risk factors (i.e. hypertension, diabetes, obesity, smoking) and the incidence of postoperative complications. However, patients with hyperlipidemia were found to be associated with significantly lower FMD% values as measured by BART.

In a clinical setting, non-invasive techniques assessing vascular function need to meet certain criteria in order to be established as practicable tools. They need to be reproducible and relatively easy to administer before they are recommended for widespread use. The predictive value of non-invasive vascular function measurements concerning perioperative morbidity and mortality has been controversial over the last years. Especially in non-cardiac surgery where there is a growing need for risk prediction, non-invasive techniques have yet to be established as useful tools in improving clinical outcomes. An increasing population of patients with cardiovascular risk factors (i.e. metabolic syndrome) serves as a counteracting force towards this dilemma. The success of either technique (BART and DTM) will depend on their ability to reliably measure an individual’s vascular function and to be added to the standard preoperative cardiovascular examinations thus refining therapeutic strategies which, in turn, will have a positive effect on postoperative outcomes.

BART studies have shown impaired vasodilatory responses in patients with cardiovascular risk factors such as hypertension [14,15], diabetes mellitus [16], hypercholesterolemia [17], and smoking [18]. In asymptomatic patients, a prospective study investigating DTM suggested that this method correlated with certain risk factors identified in the Framingham Risk Score [19]. In our study, DTM variables trended toward an association between lower BART and DTM values in patients with risk factors and higher risk scores described by: hypertension requiring therapy, diabetes, hyperlipidemia requiring statin therapy, Lee Cardiac Risk Index ≤2, and ACC/AHA Risk Score ≤2, but this failure to reach statistical significance should not preclude further investigation of these non-invasive techniques.

### BART – Strengths and weaknesses

One of the strengths of BART is that it is non-invasive and its repeatable use is applicable for monitoring the progress of atherosclerosis, especially in cardiac patients. In a prospective trial (N = 135), FMD was the strongest predictor of re-stenosis in patients undergoing stent implantation [20]. Although a large study (N = 444) suggests that measures of vascular reactivity do not have additional prognostic use in patients at high risk [6], the ability of FMD to monitor vascular function in response to therapy has been described in the literature [21-23]. A limitation of BART is its technical challenge with a significant learning curve to achieve high quality and consistent performance. Preparation and proper positioning of the patient and the sonographer ensure ergonomic comfort while minimizing both stress-related fatigue during the scan period and error [7]. Furthermore, inconsistencies in the published studies [24-27] highlight some of the difficulties of applying FMD technique in the setting of diabetes. Other confounders of FMD include arm length, sex, and postprandial state. Thus, comparison between groups of patients should use standard experimental conditions and, if possible, ensure that baseline vessel diameter and baseline blood flow are similar [28]. In our study we had similar conditions in both laboratory rooms when testing BART and DTM, patients were in the fasting state, and baseline vessel diameter and baseline blood flow were similar among the study population (3.9 ± 0.8 mm and 176.7 ± 51.7 cm/sec).

### DTM - Strengths and weaknesses

The advantage of DTM is that ultrasound is replaced by measurements of fingertip thermal changes; thereby, providing a technique that is simpler and more accessible as a point of care test (POCT). It facilitates research into the perioperative kinetics of vascular reactivity following surgery, especially in the postoperative period where patients are often restricted to the postoperative recovery area (e.g. intensive care unit or surgical floor). In a recent study, a low DTM signal was found in patients with certain cardiovascular risk factors (abdominal obesity, smoking) [29]. Another study demonstrated that fingertip thermal response as measured by DTM was inversely related to increasing cardiovascular risk independent of age, sex and other cardiac risk factors [9]. Reactive hyperemia after a period of upper arm ischemia is a physiologic response of the vasculature, depending on an endothelium-derived nitric oxide release, resulting in a rapid increase in blood flow and temperature [30,31]. DTM, utilizing this predominantly microvascular response, may be a useful point-of-care tool for the assessment of vascular function perioperatively. Furthermore, it may serve as a contributory marker of postoperative vascular function that has been reported to be impaired by systemic inflammation [32].

### Limitations

There are differences in physics between the two techniques with BART measuring vascular diameter and blood flow and DTM measuring temperature as a surrogate marker of blood flow. However, both techniques utilize the principle of reactive hyperemia after a period of ischemia, which is a known physiologic response of the vasculature and endothelial system resulting in rapid increases in both local blood flow and temperature [30,31,33,34].

DTM is highly dependent on ambient room temperature and the adjustment of the temperature probe to the surrounding condition. A stable equilibration time is required in order to avoid a temperature drift throughout the test that would have an impact on TF and TR. Also, vasoconstriction results from a cuff placed too tightly around the arm leading to false TF and TR values. This is not the only effector and other contributing factors may come from a cold fingertip (<27°C), a stressor like the white coat effect [35,36], a myogenically mediated vasoconstriction, a rise in intravascular pressure, or even a direct damming of venous outflow and capillary outflow obstruction [37]. Furthermore, the extent a neurovascular response is involved in the measurement of reactive hyperemia remains unclear. Infrared imaging of the control hand during DTM testing revealed this phenomenon, which possibly leads to blunting of the temperature response to reactive hyperemia. Evaluation of the temperature data of the left finger, functioning as a control, might give additional value to observation although its interpretation has not been fully understood.

A further limitation of our study is that the sample size of our prospective, observational study is quite low. One of our pilot studies [38] investigating BART in a surgical population identified an optimal sample size of N = 165 patients is required to have adequate power for identifying the predictive value of BART for postoperative complications in future studies. Unfortunately, we were not able to achieve this sample size in our study.

## Conclusion

To our knowledge this is the first study comparing the utility of two non-invasive techniques (BART and DTM) for the preoperative assessment of vascular function. Impaired vascular reactivity as measured by BART was associated with the incidence of hyperlipidemia but not with other preoperative cardiac risk factors or the incidence of postoperative complications. Although, non-invasive techniques assessing vascular function warrant further refinement, we conclude that BART and DTM are useful diagnostic tools for assisting preoperative optimization strategies aimed at improving vascular function in patients undergoing major surgery.

## Competing interests

The authors declare that they have no competing interests.

## Authors’ contributions

RS: Study design/subject recruitment/data collection/data analysis/scientific input/manuscript writing/reviewing. VS: Data analysis/scientific input/manuscript writing/reviewing. AS: Subject recruitment/data collection/data analysis. JA: Subject recruitment/data collection/data analysis. MH: Statistical analysis/scientific input/manuscript writing/reviewing. RJM: Scientific input/manuscript writing/reviewing. BR: Study design/data analysis/scientific input/manuscript writing/reviewing. JH: Scientific input/manuscript writing/reviewing. All authors read and approved the final manuscript.

## Pre-publication history

The pre-publication history for this paper can be accessed here:

http://www.biomedcentral.com/1471-2253/14/47/prepub

## Supplementary Material

Additional file 1 Appendix. Click here for file
