# Supplementary material for: Brachial artery reactivity and vascular reactive hyperemia for preoperative anaesthesia risk assessment – an observational study
Source: BMC Anesthesiol. 2014 Jun 21;14:47. doi: 10.1186/1471-2253-14-47 (PMC4071153; doi:10.1186/1471-2253-14-47)
Supplement: Additional file 1 — Appendix. [file 1471-2253-14-47-S1.doc]

Appendix

Adverse Event Categories and Definitions

1. Cardiac Events
   1. Myocardial ischemia without myocardial infarction^1-4^

Definition: Myocardial oxygen delivery inadequate to meet myocardial oxygen demand without causing myocardial injury.

Diagnosis: Clinical impression and two or more of the following: Angina; EKG ST segment depression 1mm or more compared to baseline EKG in 2 or more contiguous leads; EKG inverted T waves compared to baseline EKG in two contiguous leads; new dysrhythmia or BBB; Unexplained hemodynamic instability; Physical documentation of myocardial ischemia.

- 1. Myocardial ischemia with myocardial infarction^2,4-6^

Definition: Myocardial ischemia resulting in myocardial injury.

Diagnosis: Clinical impression supported by: EKG ST segment elevation of 1 mm or more; New Q waves on EKG; Serum CPK-MB fraction equal to or greater than 5%; A Serum Troponin-1 of 1.4% or higher (excluding renal failure patients); Physician documentation of myocardial infarction.

- 1. Dysrhythmias and conduction abnormalities^7-8^

Definition: Any cardiac rhythm or condition pattern other than normal sinus rhythm, or a new or exaggeration of preexisting dysrhythmia or conduction defect.

Diagnosis: Evidence of above definition on EKG tracing; Physician documentation of dysrhythmia and/or conduction abnormalities.

- 1. Congestive heart failure^9-10^

Definition: Decreased myocardial contractility resulting in signs and/or symptoms of pulmonary or systemic congestion.

Diagnosis: Clinical impression supported by: Evidence of systemic venous congestion and/or pulmonary congestion on physical exam; Chest x-ray evidence of pulmonary edema and/or cardiomegaly (cardiothoracic ratio >0.5); Indications of right, left, or biventricular myocardial failure via pulmonary artery occlusion pressure (>18mmHg) and/or echo cardiography ejection fraction <40%; Physician documentation of congestive heart failure.

- 1. Postoperative Vasopressors

Definition: Any postoperative patient who require vasopressor support by continuous IV infusion.

Diagnosis: History of/or presently receiving vasopressors postoperatively.

- 1. Cardiac arrest with successful resuscitation

Definition: Sudden cessation of cardiac function with disappearance of arterial blood pressure

1. Respiratory Events
   1. Prolonged Intubation

Definition: Patient intubated from the end of the operation for over 24 hours.

Diagnosis: Evidence of patient being intubated for over 24 hours in chart.

- 1. Reintubation Postoperation

Definition: Any patient who postoperatively was extubated within the 10-day period of monitoring and has to be reintubated for any reason within the monitoring period

Diagnosis: Evidence of reintubation during the monitoring period from the patient’s chart; Physician documentation of reintubation.

- 1. Acute respiratory distress syndrome (ARDS)^11-14^

Definition: Noncardiogenic pulmonary edema

Diagnosis: PaO_2_/FiO_2_ less than or equal to 200; CXR = Bilateral pulmonary infiltrates; No clinical evidence of atrial hypertension; Pulmonary artery occlusion pressure less than or equal to 18 mmHg; No cardiac explanation for impaired oxygenation; Physician documentation of ARDS

- 1. Hypoxemia^15-18^

Definition: Deficient oxygenation of tissue

Diagnosis: PaO_2_/FiO_2_ less than or equal to 300 or pulse oximetry <90; Physician documentation of hypoxemia

- 1. Pneumonia^15-18^

Definition: Lower respiratory tract infection

Diagnosis: Clinical impression along with 2 or more of the following: Abnormal breathing sounds compared to baseline; Temperature greater than or equal to 38°C; New productive cough; positive sputum cultures; CXR documentation of atelectasis or new infiltrates; Physician documentation of pneumonia.

- 1. Acute Respiratory Failure^19^

Definition: The inability to independently support one’s ventilatory and/or oxygenation needs without medical intervention

Diagnosis: Clinical impression supported by: Progressive dyspnea; PaO_2_/FiO_2_ less than or equal to 300; Pulse oximetry less than or equal to 90%; Physician documentation of respiratory failure

1. Wound Healing Event
   1. Wound Infection^20^

Definition: Wound healing delayed by microbial infection

Diagnosis: Purulent material draining or aspirated from wound and/or a positive culture from wound. Physician documentation of wound infection in chart.

- 1. Sepsis^21^

Definition: The presence in the blood of pathologic microorganisms or their toxins

Diagnosis: Clinical impression along with the following: Signs of systemic response to infection, tachypnea, tachycardia (>90 beats/min), hyper or hypothermia (>38.4°C or <35.6°C), physician documentation of chart.

1. Surgical Events
   1. Prolonged Air Leak^22^

Definiton: Air leak from bronchopleural fistula, from denuded visceral surface of the lung, or other origin requiring continuous suction under water seal and/or other nonsurgical maneuvers.

Diagnosis: Physician documentation of air leak for more than 5 days after surgery.

- 1. Esophageal Leak^23^

Definiton: Anastomotic dehiscence post-esophagectomy requiring surgical intervention

Diagnosis: Clinical signs and symptoms of a leak confirmed by a radiological water-soluble contrast study of the esophagus.

- 1. Reoperative Event

Definition: Patient undergoes an invasive procedure not limited to the operating room secondary to a complication resulting from the initial elective surgical procedure

Diagnosis: Physician documentation of invasive procedure in the chart
